# LIBRA: A simulation-based gender equality training programme for student leaders in higher education

**DOI:** 10.1186/s41077-025-00404-9

**Published:** 2026-04-16

**Authors:** Michelle Kirrane Scott, Siobhán Lucey, Mide Power, Siobhán M. Lucey, Claire Condron

**Affiliations:** 1 RCSI University of Medicine & Health Sciences, Dublin , Ireland; 2 Department of Economics, University College Cork, Cork, Ireland; 3 Department of Sociology, Trinity College Dublin, Dublin, Ireland; 4 Cork University Dental School and Hospital, University College Cork, Cork, Ireland

**Keywords:** Gender equality, Student leaders, Simulation-based education

## Abstract

**Background:**

Training is required to build the capacity of future leaders to dismantle disadvantage, influence change and strengthen diversity and inclusion across higher education institutions. The LIBRA pilot project uses a novel simulation-based approach to equip student leaders with skills to address biases and promote gender equality.

**Methods:**

Kern’s Curriculum Framework was utilised to systematically design a simulation-based gender equality training programme for students, employing a six-step approach that included problem identification, a comprehensive needs assessment using a mixed methods approach, and development of educational objectives and strategies. The LIBRA programme was piloted by 19 student leaders across two sites. The programme was evaluated by assessing participants’ pre- and post-event confidence to deploy skills and techniques which foster gender equity in post-secondary settings. Participant feedback was also collated via an online questionnaire.

**Results:**

Students’ confidence in their ability to communicate, be actively aware and challenge gender equality matters increased post-intervention. They found the programme engaging, interesting, accessible, and relevant. Students felt comfortable participating in the training and found that the feedback provided to them was useful. The majority of students strongly agreed that simulation is a valuable approach to support the acquisition of gender equality competencies.

**Conclusions:**

Within the LIBRA pilot project, a simulation-based approach to tackling gender inequality was positively received by student leader participants. The LIBRA programme can be adapted for use in local contexts to create a co-operative simulated learning experience for participants. Further research is required to evaluate the impact and value of such training programmes within the context of wider Equality, Diversity and Inclusion (EDI) initiatives.

## Introduction

Historically, higher education institutions (HEIs) have been patriarchal in design and structure. These institutions continue to be both gendered and gendering organisations despite their potential to be significant forces for advancing gender equality, diversity, and inclusion, not only in the context of higher education but also in society at large [1]. Zippel and Ferree [2] describe the unique duality of universities as “*sites of both elite knowledge production and reproduction of intersecting gendered inequalities”*. The representation of women and gender-diverse individuals in leadership positions within HEIs remains disproportionately low, with inadequate policies and support systems perpetuating unequal treatment and barriers to advancement. Across the European Union, women constitute 48.1% of doctoral graduates (ranging between 40% and 60% in the majority of countries) and constitute 43.2% of academic staff [3]. However, women represent only 26.2% of Grade A academic roles (equivalent to full professorships) and 23.6% of heads of HEIs [3].

These unequal systems and cultures are prevalent across the higher education sector. For example, in academic science and medicine, Coe et al. [4] reported a lack of awareness and education among leaders and practitioners about the institutional and systemic barriers impeding gender equality. Limiting the progression of women in these fields impacts the potential for global medical advancements [4, 5]. Samra [6] called for the implementation of a gender-responsive approach in medicine to examine and transform the unequal, patriarchal foundations of the discipline. The urgent need to implement interventions at all levels of academic medicine—from organisational governance to individual awareness—to realise gender equality is well documented [4, 5, 7]. Among the interventions recommended, Coe et al. [4] highlight the need for inclusive leadership across all institutional levels; professional development interventions to educate and upskill leaders and faculty in realising gender equality; and the integration of Equality, Diversity and Inclusion (EDI) training into medical curricula to establish diversity and inclusion as an institutional and cultural norm.

A training need has been identified to increase awareness and nurture competencies for student leaders to actively address gender inequality, and such training can create an important impact on the wider HEI community [8]. Educational initiatives should promote critical thinking, challenge biases, foster an understanding of complex issues and encourage skill development. Multiple gender equality-based training initiatives for students have been designed and evaluated in second- and third-level education settings, e.g., didactic teaching, face-to-face collaboration projects, site visits, case studies and coaching [9–11]. However, there is a dearth of research conducted to date examining the application of simulation-based education (SBE) for gender equality training. Simulation is an immersive, experiential learning technique for practice and learning that amplifies real experiences with guided encounters, evoking or replicating substantial aspects of the real world [12, 13]. The SBE framework, comprising pre-briefing, event, and debriefing, supports constructivist principles by engaging learners in immersive experiences [14].

SBE can also be understood through a sociocultural lens, highlighting the role of collaborative learning. In SBE, the pre-briefing event and debriefing phases not only engage students in experiential learning but also embed them within a social framework where learning is co-constructed through interaction with peers and facilitators [15]. SBE develops attention, intuition, perception categorisation, discrimination, acquisition, search, thought, suggestions, decision-making, knowledge and linguistic processes [12, 13, 16–18]. A large-scale narrative analysis conducted with health and social care professionals illustrated the value of SBE as a learning approach, providing a realistic, holistic and transformative opportunity to develop knowledge, skills and attitudes that are translated into practice. Participants emphasised that the ability to make mistakes in a safe environment was particularly powerful, facilitating ongoing reflection and improved practice in future real-life contexts [19]. Chernikova et al. [18] and Cleland et al. [15] provide meta-analyses and case studies on the sociocultural complexities of simulation-based education. These studies demonstrate how simulation can enhance learning outcomes and address gender biases across different higher education domains. SBE therefore offers an important opportunity to approach training in gender equality differently, by engaging learners in real-life-based situations in ways that more traditional training methods do not. In Ireland, the Higher Education Authority Reports [20, 21] review progress in gender equality in Irish HEIs and highlight areas to be addressed to continue progression towards gender equity.

### Theoretical framework

Dhatt et al. [22] propose a new vision for gender-transformative leadership (GTL) in global health. A gender-transformative leadership (GTL) framework was used to guide this study [22]. GTL strives to address and overcome gender inequality in the higher education sector globally. This framework highlights the need to concurrently challenge gender inequality at all levels, from unequal systemic and cultural inequity to interpersonal social norms. The GTL framework is rooted in intersectionality [23], emphasising the importance of understanding the ways in which gender intersects with other social locations, such as race, class and sexuality, and how gender inequality intersects with different forms of oppression, such as racism, classism and heterosexism. The GTL framework is based on the belief that work to drive gender equality must be holistic, recognising the interactive and intersecting institutional power structures that perpetuate oppression.

The purpose of our project is to future-proof gender equality in HEIs by working with leaders of the future to address biases and accelerate culture change. Grounded in the overarching GTL framework, we drew on Myra Marx Ferree’s [24] ‘Knowledge, Desire, Ability’ (KDA) Theory of Change, as recommended by UN Women’s gender equality training guidelines [25]. The application of this approach to support gender equality training requires all three components: knowledge of gender equality principles and gender inequalities; the desire and motivation to implement change; and the tools and competence to bring about change.

### Aim & objectives

The overall aim of the LIBRA research project is to design and evaluate a simulation-based training programme to teach skills that address gender bias and raise awareness of inclusive student leadership behaviours in realistic contexts. To achieve this, the LIBRA research project was guided by five principal objectives:

1. To explore student leaders’ perceptions and experiences of gender and gender equality in higher education;
2. To identify competences required to promote gender equality;
3. To develop a comprehensive skills framework, detailing learning outcomes and a comprehensive training programme to support gender equality leadership skills;
4. To design and create a gender equality leadership training programme for students in higher education using experiential learning;
5. To evaluate the effect of the SBE gender equality programme.

## Methods

To address these five objectives, the research design consists of two key stages, as follows:

(1) The development of the LIBRA gender equality leadership training programme for students in higher education, using simulation-based experiential learning;

(2) Piloting the LIBRA programme at two sites to assess the efficacy and impact of the programme.

The LIBRA Project was undertaken as a collaborative initiative across three HEIs (a healthcare professions-focused university, a large university with a broad range of teaching programmes and a drama-focused department in a third HEI).

### LIBRA curriculum development

Kern’s 6-step Curriculum Development Framework was applied to design and implement a simulation-based gender equality training programme for students [26].

#### Kern’s step 1—problem identification and general needs assessment

**Fig. 1:**
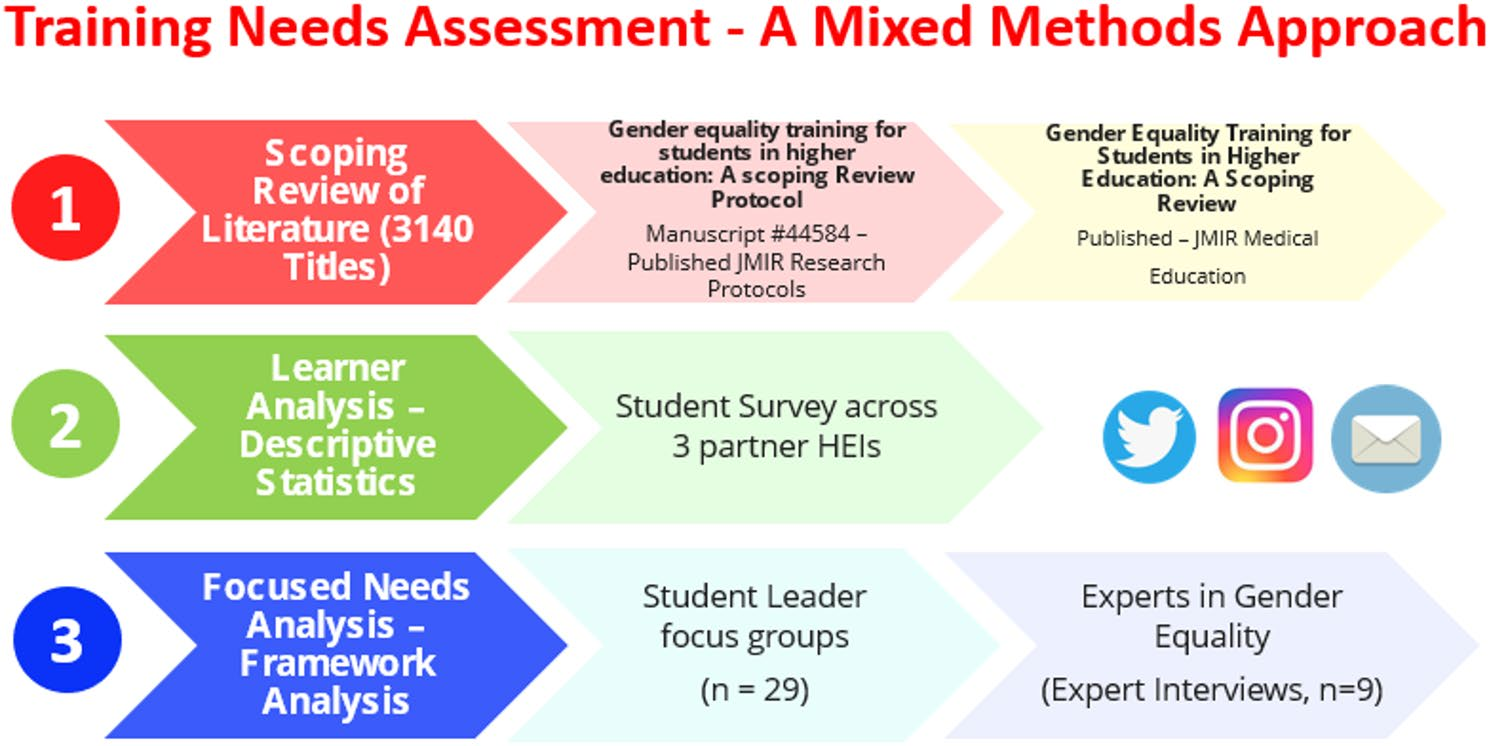
A mixed methods approach to training needs assessment This diagram presents the mixed methods approach employed to carry out a comprehensive needs assessment of students’ suggestions, ideas and needs for gender equality training. This approach combines qualitative and quantitative research techniques to enhance the understanding of skill gaps and thus inform the development of targeted educational interventions

A needs assessment was conducted to explore students’ perceptions and experiences of gender equality and inequality, and to identify gaps between the existing competencies of student leaders and the skills required to support gender equality (Fig. 1). A scoping review was conducted to identify and map best practice regarding gender equality training for university students [27]. This informed the design of empirical data collection, complemented by the Irish Higher Education Authority’s Gender Equality Action Plan [20].

#### Kern’s step 2—targeted needs assessment

Thirty-six student leaders (Table 1 in Appendix A) completed a survey designed to (1) assess their experiences and thoughts on gender equality/inequality in daily life and in higher education, and (2) to ascertain students’ experiences and perceptions of gender equality and gender balance in their course of study. Eligible participants included Students’ Union officers, class representatives and club/society committee members across the three collaborating universities.

Six focus groups were carried out with twenty-nine student leaders, who discussed their experiences and perceptions of gender and gender equality as students in higher education. The needs assessment also included nine interviews with experts in gender equality and leadership from a variety of fields, such as academia, the corporate sector and non-profit organisations. The interviews focused on participants’ experiences in promoting gender equality and leadership and considered the core competences required to foster gender equality. The student focus groups and expert interviews were facilitated by experienced members of the research team.

#### Needs assessment - data analysis

The survey data was analysed using descriptive statistics. Framework analysis was employed to identify, describe, and interpret key patterns in the focus group and interview data guided by the aims of the study [28, 29].

#### Needs assessment—outcomes

Survey respondents reported that they encounter gender stereotypes or sexism monthly in lectures/seminars and on campus, which increased to a daily or weekly occurrence in the settings of a clinical placement, a professional internship, a university sports event and a student union event. With respect to gender equality relating to gender-diverse and transgender individuals, students reported a low level of equality in course materials and student leadership roles. However, they did report a high level of gender-diverse equality in teaching staff and supervisors’ treatment of students and students’ treatment of each other. Students agreed that in situations where they held a leadership role, it was easier for them to both promote and be more aware of gender and gender equality. When asked to rank the importance of six gender equality skills, the skill of ‘*Understanding*,* and defining gender equality principles’* was ranked 1 st whereas the skill of ‘*Challenging gender stereotypes and sexism among their peers*,*”* was ranked in 6th position. Across the gap analysis phase, through focus group discussions and completing the survey, participants identified specific contexts and situations where they have encountered gender inequality and, thus, where gender equality leadership skills would be beneficial to address and challenge such inequalities (Table 2 in Appendix A).

Across the focus groups, students’ perceptions and understanding of gender equality was consistent, emphasising, gender balance and representation, equality of opportunity and resources, as well as equity, tailored supports and equality of outcome for people of all genders. Students described gender inequality as a systemic issue, and a need to address the societal structures that perpetuate inequalities to remove barriers and realise gender equality. Participants highlighted the importance of allyship and a collective responsibility to bring about gender equality, where men leverage their power and resources to support gender equality. Participants consistently highlighted the importance of promoting gender equality across the gender spectrum and not just focusing on a binary understanding of gender as man or woman. In relation to students’ experiences of gender equality and inequality, participants discussed gendered microaggressions, stereotypes and derogatory behaviour they had encountered and witnessed, and gendered roles they had been assigned, in campus environments, work placements, social settings, sports clubs and on social media. Many students described preferential treatment of men over women in placement settings and in the classroom, for example, giving men more learning opportunities. However, two male participants discussed preferential treatment of women by some female staff members. Students discussed a lack of awareness about gender diversity in society, and emphasised the importance of giving people the skills, language and tools to promote equality - and address inequality - across the gender spectrum.

These real-world examples enabled us to create authentic narratives, situations and atmospheres for SBE scenarios.

#### Kern’s step 3—goals and objectives

The needs assessment outcomes were used to define a competency-based KDA Framework (Table 3 in Appendix A). Supporting quotes from focus group and interview participants are provided in Table 4 in Appendix A. The KDA Framework informed the learning objectives for the LIBRA programme, as follows:

1. Knowledge: Understand gender equality in educational and social contexts.
2. Desire: Practice reflection to gain an insight into personal implicit biases and how these may impact our behaviours when interacting with peers and educators.
3. Desire: Demonstrate the attributes (e.g. confidence, responsibility) to actively promote gender equality in a student leadership role.
4. Ability: Actively address gender inequality among peers and educators in a student leadership role.
5. Ability: Employ communication competences appropriate to context to promote gender equality and challenge inequality.

#### Kern’s step 4—educational strategies

The LIBRA simulation-based programme consisted of five simulated scenarios, which included pre-encounter content, group-learning interactions with simulated participants (SPs), peer feedback and post-encounter reflection and debrief. Scenario themes included microaggressions, power dynamics, allyship, gender stereotyping, gender diversity, use of correct pronouns, sexual harassment and bystander intervention. Scenario contexts included lectures, academic group projects, clinical placement, student club/society committee meetings and a student party. Student participants were directed to asynchronous training resources to facilitate self-directed learning pre-workshop. A ‘Train the Trainer’ pack was developed to support faculty in delivering this programme. Drama students, actors and SPs were trained over two days to portray the roles scripted in the scenarios. Table 5 in Appendix A provides a summary of each of the 5 scenarios with the full details of the LIBRA programme content outlined in Appendix B: LIBRA Programme Resources and available here.

#### Format of the scenario progression and scenario debrief

Simulated training scenarios were designed for the student to take on the protagonist role and interact with the trained SP. In accordance with the principles of the forum theatre as applied to simulation training [30, 31], facilitating the scenarios allowed students to ‘pause’ a simulation scenario at any moment. This approach empowered new students to ‘tap in’ or existing participants to ‘tap out,’ facilitating the exploration of different strategies and perspectives within the scenario. This dynamic interchange was designed to enable participants to experiment with various approaches to problem-solving and decision-making in a controlled environment, fostering a deeper understanding and engagement with the material.

Debrief discussion templates for each scenario were designed for facilitators, to guide reflection on the perspectives of the participant(s), the SP(s), and students who participated as observers. The debrief space provided an opportunity to engage in peer feedback and to reflect on actions employed during the scenario. Shared discussions identified ‘what worked well’ and ‘what different approaches might work’ - providing an opportunity for students to put the KDA into practice and identifying skillsets for stepping into real life situations.

#### Psychological safety

*Structured Pre-briefing sessions*: We incorporated structured pre-briefings at the start of each session. These sessions were designed to establish ground rules, clarify learning objectives, and offer participants the opportunity to raise any concerns. This facilitated the creation of a psychologically safe and “brave” space conducive to open and respectful participation.

*Support resources embedded in the learning environment*: In addition to signposting institutional support services (e.g., through our student services team), we ensured that trained facilitators or mental health professionals were present during sessions. This provided participants with immediate access to support if needed.

### LIBRA programme pilot

#### Kern’s step 5 - implementation of the LIBRA programme

The programme was piloted at two institutions (RCSI, UCC), with nineteen student participants. Pre-training content was supplemented by an introductory workshop on the morning of the in-person training day. Participating students were aged 20–34 and evenly split between undergraduate and postgraduate programmes, with most studying health sciences (57.9%) and smaller proportions in Arts and Humanities, Engineering, Business, Law, and Science. Respondents identified across a range of gender, sexual-orientation, and ethnic categories; full demographic details are provided in Table 6 in Appendix A. The target audience comprised student leaders (18 years and older), defined as Students’ Union leaders, class representatives, and student club and society committee members.

The pilot training sessions were enriched by the diverse expertise of the faculty, which included a blend of simulation educationalists and EDI specialists from the partnering institutions. This combination of multidisciplinary expert guidance created a robust platform for students to develop both their practical skills and their appreciation for diverse perspectives and inclusive practices.

#### Kern’s step 6 - evaluation and iterative improvement

A pre- and post-intervention confidence instrument was administered to learners. A QR code was provided at the end of the session for post-intervention questions and evaluation feedback. The instrument design was informed by published evaluation tools for gender equality training [32] and was piloted prior to use [33]. Evaluation feedback was collected via an online questionnaire, which included an option for free-text responses. Verbal feedback was gathered from faculty members, supplemented by detailed field notes taken by the researchers.

Programme evaluation was also completed by the SPs who were asked to comment on the training resources provided to them, the processes of carrying out the LIBRA pilot and the impact of the LIBRA programme.

## Results

**Fig. 2:**
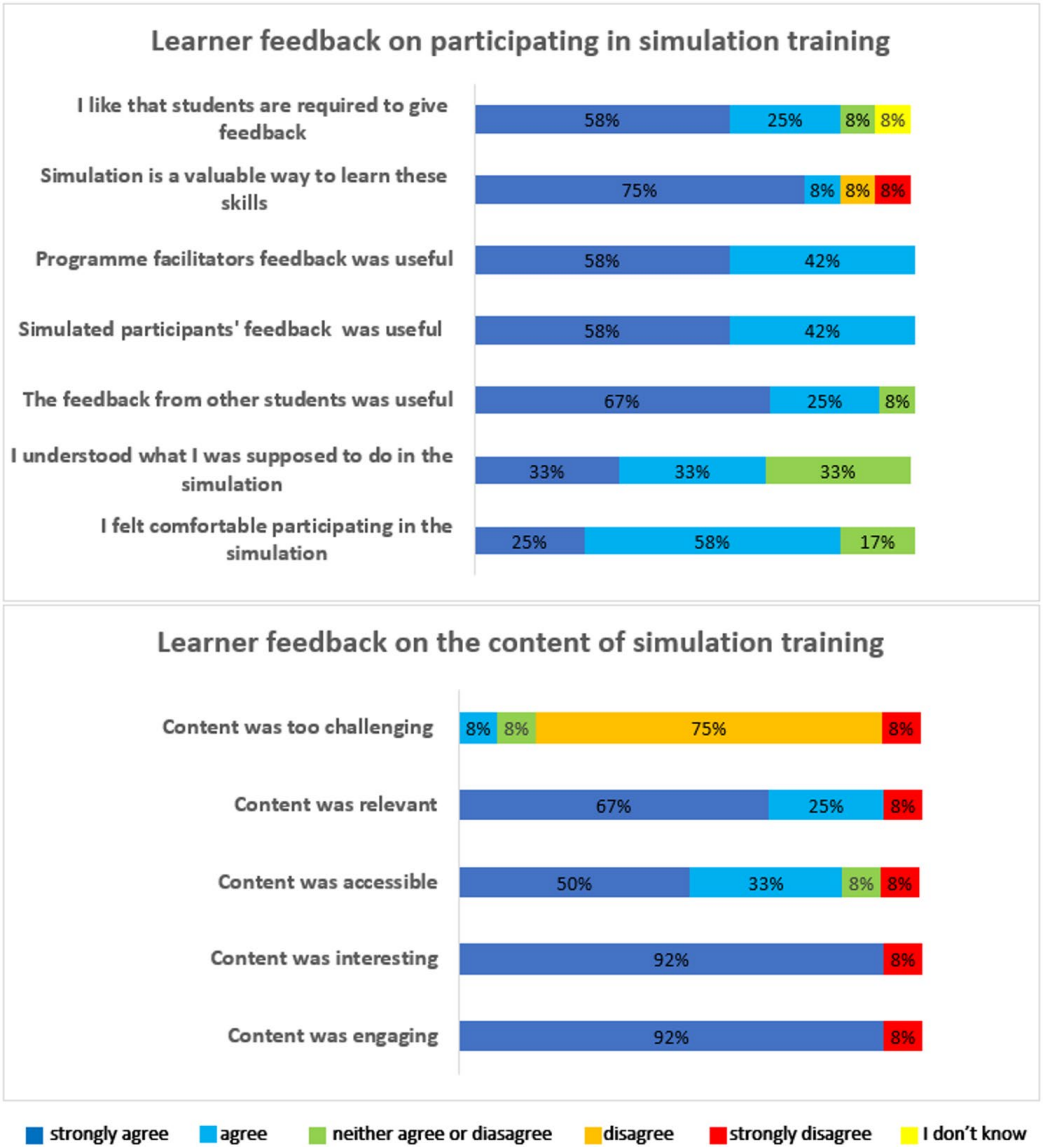
Student evaluation of the LIBRA programme This figure presents the student evaluation of the LIBRA programme. Post-event evaluation surveys were completed by participants via a 6-point Likert scale. Data are expressed as percentages of the number of students who chose that point on the scale

Pre- and post-intervention confidence results are summarised in Table 7 in Appendix A. Using a 6-point Likert scale for the question, *“I am confident in my ability to…”* students were asked to rate their level of agreement on ten gender equality statements. Students reported increased confidence in their ability to understand, communicate, be actively aware of and challenge gender equality post-intervention (proportion of agree/strongly agree) for each statement, except for one, ‘challenging gender inequality amongst peers’. Illustrative excerpts from the qualitative feedback are presented to contextualise the quantitative findings; however, no formal coded or thematic analysis was undertaken (Table 8 in Appendix A). The students strongly agreed that the content of the programme was engaging, interesting, accessible and relevant and that it was not too challenging (Fig. 2). The students agreed that they felt comfortable participating in and understood what they were supposed to do during the simulated scenarios. The students strongly agreed that the various types of feedback provided to them were useful and that SBE is a valuable way to learn the skills necessary to promote gender equality competencies in leadership positions.

## Discussion

The LIBRA project, named for the scales of balance and justice, aims to future-proof gender equality by working with the leaders of the future (men, women, non-binary student leaders) to address inequity in the higher education sector. Following simulation training, participants reported more confidence in their gender-equality leadership skills. This was mirrored in the feedback provided by students and SPs on the course content and their experience of learning. A process-oriented approach was taken to the design of these interventions from the outset with a comprehensive needs assessment and participatory design methodology used for scenario writing. This was important to ensure that the content was reflective of student experiences and authentic gender equality issues that they might face.

### Theoretical framework: gender transformative leadership

Theoretically grounded in GTL and empirically grounded in a KDA framework, the LIBRA programme was designed to enable participants to increase awareness of their own biases, examine their own positionality as individuals and leverage their leadership roles to challenge the cultural biases and norms in academic environments that reproduce inequality [22]. Through training delivery, we emphasised that the competences taught could be applied in students’ own academic and social circles as students, but we also aimed to provide skills and knowledge that they could bring to their future professional careers. Student leaders were chosen as a target group, owing to their status among peer groups, and consequently, their influence on the social norms around them. LIBRA is well placed to support the implementation of a gender-responsive approach in education; to examine the patriarchal cultures, practices and prejudices embedded in the sector; and to upskill student leaders in bringing about a culture of gender equality and fairness. The programme was also designed with a temporal lens, aiming to provide leaders of the future with the understanding and skills to promote gender equality at the organisational, institutional and systemic levels.

### Theory of change: Knowledge, Desire & Ability

As argued by Ferguson [25], it is not possible to create a singular overarching theory of change for training for gender equality. Rather, training must be developed in relation to the context in which it will be deployed. Ferguson [25] contends that trainees’ engagement in active change work in their organisations will be improved if their training increases both their knowledge and curiosity (desire to learn) while providing advocacy skills or access to different networks of knowers (ability to challenge gender). As Ferree [24] argues, the first step towards improving gender relations in organisations involves identifying the features of the system that are producing these inequalities.

The LIBRA KDA framework draws on multiple studies examining leaders’ roles in promoting gender equality. Noteworthy features of such leadership approaches include understanding, continual learning and championing gender equality issues to enhance understanding of others (Knowledge); making time for self-reflection and self-awareness of one’s own privilege and biases; possessing the courage to take risks (Desire); communicating dedication to equality principles; challenging direct and indirect discrimination; employing care and empathy in gender equality efforts; and collaborating with others in gender equality initiatives (Ability) [34–38]. Furthermore, Rosener [39] discusses the importance of facilitating diversity in leadership styles, whereby female leaders should not have to fit the traditional male ‘model’ of leadership and can lead successfully to use the attitudes, skills and habits they have developed as women.

The theory of change construct also proposes methods of evaluation across the three domains of knowledge, desire and ability, including comparative learning needs assessment before and after training, interviews, and questionnaires, in addition to longitudinal measurement of participants’ abilities via qualitative approaches [25]. The LIBRA Programme incorporates elements of all these evaluation approaches, except for the long-term follow-up of participants.

We recommend that future iterations of the LIBRA programme incorporate longitudinal assessment to evaluate the impact of the training programme over time and ascertain whether student leaders’ post-intervention level of confidence and perceived competence in promoting gender equality are sustained. In addition, we acknowledge that training by itself cannot bring about gender-transformative change but should be embedded within a broader set of measures and actions to influence change [25]. The UN Women’s Training Centre [25] has recommended that training programs should be anchored within a larger process of institutional gender mainstreaming.

### Benefits of the simulation approach

SBE approaches create conditions that optimise learning by enabling participants to experience, in real time, the consequences of their decisions and actions. Interestingly, there appears to be limited evidence available with respect to the use of simulation in gender equality-based education. We argue that SBE presents an exciting and under-utilised opportunity for innovation in gender-equality training. The results of the needs analysis reflect previous findings from the literature in relation to college students’ experiences of gender inequality, such as stereotyping, microaggressions, sexual harassment, power dynamics between students and staff, and preferential treatment of male students [40–43]. In addition, the contexts represented in the scenarios are also representative of the environments discussed in prior publications on gender inequality and harassment, for example, classrooms, college campuses, societies and sports clubs and medical practice placements [40, 41, 43]. A study by Beavers et al. [44] used an alternative simulation method (virtual reality) to address racism in a healthcare setting. The authors found that such interventions “showed potential as educational interventions to support participants in learning to take action to address racism.” This illustrates the opportunity to adapt scenarios across other types of simulation methods. Dieckmann and Nirula [45] suggest that simulation educators should engage in meaningful scholarship to understand more about the impact of simulation in exploring Equity, Diversity, Inclusivity and Accessibility (EDIA) topics.

The results of our pilot programme indicate that participants generally gained confidence in their communication skills and their ability to recognise gender inequality issues. However, following participation, we note with interest that students were more confident in challenging their close friends than their peers with respect to gender inequality issues. This phenomenon may have occurred because of an increase in the participants’ conscious awareness of the barriers and challenges that must be faced in social settings when addressing gender inequalities. It is likely that greater reticence will exist when one interacts with peers rather than in the comfort of one’s close circle of friends. This finding is in line with Balliet et al. [46] whose study showed that people are more likely to directly call out or confront misconduct by a close friend and, conversely, will use social sanctioning strategies such as gossiping about people with whom they did not have a close relationship.

### Multidisciplinary acceptability

Importantly, the LIBRA initiative was not targeted at any one sector or discipline within the participating HEIs. We argue that this is a unique attribute of the programme and that it is likely to increase acceptability and engagement by participants and existing university leaders by transcending discipline-specific issues. The simulation scenarios in the LIBRA programme are easily transferrable across various sectors of higher education, e.g., group project work, lecturer‒student interactions and bystander intervention techniques. For example, one of the simulated scenarios was written in a modifiable style, allowing it to be carried out as a scenario involving a practice placement or a professional internship. It is our view that the LIBRA programme has the potential to supersede disciplinary norms because of this transdisciplinary approach. While further work is required to verify this supposition, we hold that the authenticity of the LIBRA scenarios developed with students, and for students, is likely to have greater impact within higher education organisations than discipline-specific training.

Systemic discriminatory practices in the Canadian science, technology, engineering and mathematics (STEM) higher education context have been addressed in recent work from Ruel & Tajmel [47]. Using a mixed-methods approach, the empirical findings and data analysis led to the development of an intentional EDI framework, constructed into three main pillars represented by the figure of a tree: the foundational elements (roots) built on individuals’ complexities and experiences of othering, the possible interrelationships (trunk) across various educational and professional dimensions, and a call to structural change initiatives (branches). Notably, the transdisciplinary stance adopted in the LIBRA is well aligned with the second pillar of the proposed framework. Ruel & Tajmel [47] identified a specific need to put in place EDI courses that cross faculty boundaries, which has been the very essence of LIBRA from conception to realisation.

### Limitations

The pilot studies were carried out in June. As this timeframe was outside term time, it was difficult to recruit sufficient student leaders to partake in the study. We offered vouchers to the value of €50 for participation in the course to incentivise involvement. While this enabled us to recruit nineteen participants and complete a comprehensive bi-locational pilot study, it is important to consider that students’ engagement in the simulated scenarios and in the debrief discussions might not have been at the same level without a reward.

In addition, it is important to consider the selection bias of the students who opt to become involved in the pilot studies. Many participants had previously been involved in initiatives and training in topics such as EDI, gender, gender equality and gender diversity. When rolled out more broadly, it will be important to ascertain whether the LIBRA has the same level of efficacy and is regarded as highly effective by a broader student group.

We recognise that a sample size of 19 participants limits the generalisability of our findings. We conducted this initial pilot study on a small scale to explore the feasibility of SBE for gender equality and leadership training. Further, although the same survey was administered pre- and post-intervention to the same cohort, paired analyses were not conducted because our ethical approval required full participant anonymity. This anonymity was essential for maintaining psychological safety and encouraging candid self-assessment but limited the scope of data analysis and the conclusions that could be drawn.

Students’ and SPs’ feedback was crucial to determine students’ priorities and recommendations for future SBE training. The small sample size highlights the need for more systematic planning in two key areas: recruitment and retention whilst ensuring continued representativeness. To strengthen participation across disciplines and demographics, a targeted outreach approach may be adopted. This includes partnering with student organisations—such as cultural societies, LGBTQ + groups, and disability networks—to engage underrepresented student populations and ensure messaging is inclusive and relevant.

Students’ feedback illustrated their appetite for intersectionality to be incorporated more centrally throughout the programme. The primary aim of the LIBRA programme was to establish foundational learning experiences in gender equality through simulation. While we incorporated discussion and reflection on intersectionality in the pre-brief and debrief components of the programme, students recommended creating scenarios that deal more explicitly with multiple, overlapping experiences of discrimination. Students’ feedback aligns with Choo and Ferree, who propose transformational intersectional research frameworks, recognising overlapping, systemic and relational power inequalities [48]. The adaptability and flexibility of the simulation-based LIBRA programme facilitates the feasibility of adapting training content to place intersectionality at the heart of students’ learning.

### Implications for future research

#### Intersectionality and programme adaptability

Students’ feedback demonstrated the importance of designing intersectional equality training, to comprehensively address the relational nature of overlapping experiences of systemic dominance and oppression. for example, one student’s free-text post-encounter feedback noted: “I am aware this is in regard to gender equality but I would love to see the inclusion of race, disability and so on to express that people of different backgrounds can be treated differently not only women vs. men. I think it would be great to see simulated scenarios cover issues of misgendering or violence towards men or male presenting people.” Embedding intersectionality throughout the LIBRA programme would align with contemporary guidelines for many gender equality and EDI-based policies and initiatives, emphasising a need to move away from viewing gender in isolation and toward promoting gender equality with a more diverse and inclusive lens [22, 49, 50].

Future directions for LIBRA should adapt one or more of the current scenarios to place intersectional experiences at the centre of students’ learning. Further, new scenarios could be added to the current selection within the LIBRA programme.

#### Programme adaptability for global North & South

The simulation format of the LIBRA programme was designed using a low-resource approach; thus, this training can be implemented in any setting without requiring costly technology or equipment. LIBRA can be rolled out in resource-constrained environments, ensuring that progress toward gender equality is accessible and scalable across diverse contexts.

#### Psychological safety

Throughout the training, a focus was placed on the importance of the learning environment, ensuring a psychologically safe space to learn and practice. An emphasis was placed on the opportunity to learn from, with and about one another with a shared input from both facilitators and participants. The fact that the training raised potentially challenging situations and content for all was identified and addressed at the start of training. The participants reported that they were able to freely share thoughts and perspectives without judgement. Importantly, 17% of the SPs felt that engaging in the LIBRA programme could have negative effects on students, and 17% of the students chose ‘neither agree nor disagree’ in response to the question “I felt comfortable participating in the scenarios”. Facilitators of experiential learning activities need to be continually mindful of psychological safety, ensuring motivation and accountability in learning and enacting a commitment to respecting learners and their concern through the intervention. Furthermore, transparency and guidance are needed for both SPs and students about what is expected of them in the simulated scenarios, for example, by spending dedicated time outlining specific learning objectives in advance of each scenario.

The scenarios address difficult subject matters, such as misogyny, microaggressions and sexual harassment. Although some students felt unsure or uncomfortable during a scenario, they felt that this was a rich learning experience to prepare them for encountering such a scenario in real life and provided space to reflect on their actions in a simulated scenario. Students’ feedback echoes participants’ experiences in Bearman and colleagues’ narrative analysis [19], whereby students described the challenging and, at times, overwhelming nature of engaging in emotional or dramatic situations and feeling self-conscious of making mistakes. However, there was a recurrent agreement that learning to correct their mistakes and manage feelings of discomfort in a safe, immersive environment were very valuable learning opportunities to equip them with real-life practice.

Integral to the success of the programme is creating a psychologically safe space, which was achieved in part through a forum theatre approach, encouraging students to ‘tap out’ or ‘pause’ a scenario whenever they wished to do so. The realism of forum theatre simulation has been shown to enhance engagement, evoke empathy among participants and develop listening skills [30].

Feedback from some simulated participants (SPs) and students raised valid concerns about the potential for unintended negative emotional impacts during simulation. This highlights the importance of proactively addressing psychological safety as a core element of educational design. Going forward, each scenario should include a specific and clearly worded trigger warning, provided in advance. This will enable participants to anticipate emotionally challenging content and make informed decisions about their level of engagement. Embedding trigger warnings will support ethical delivery, respect individual boundaries, and foster a more supportive learning environment.

#### Programme engagement & participant numbers

To reduce barriers to participation and maintain engagement, we aim to integrate the LIBRA programme into existing curricula wherever possible. This will help position the training as a core component of professional development rather than an optional “add-on.” Additionally, we are exploring the use of participation incentives, such as digital badges or portfolio credits, to formally recognise learner engagement and encourage sustained involvement and participant retention.

For future researchers exploring gender equality training through SBE, we propose expanding recruitment to ensure the student sample is large enough to ensure statistical representativeness of the institution’s student population. We would recommend oversampling among the student body to ensure a statistically representative sample, accounting for the likelihood of participant attrition. Finally, we would recommend testing future gender equality SBE training programmes using further prospective studies with larger number of participants and a variety of quantitative and qualitative outcome measures to comprehensively assess the impact of the training outcomes.

## Conclusion

The LIBRA pilot project developed an adaptable and meaningful gender equality training programme, based on a defined KDA framework which was co-created with students. The simulation-based education approach provided an opportunity for students to embed learning and reflexively recall appropriate actions for situations that challenge gender equality. Student leaders reported increased confidence in the gender equality knowledge and skills following participation in the program, and the simulation approach was positively received. The LIBRA programme can be adapted to create a simulated learning experience for participants, grounded in local contexts. Further research is required to evaluate the impact of simulation-based training programmes within the parameters of wider gender equality initiatives.

## Data Availability

No datasets were generated or analysed during the current study.

## Appendix A

**Table 1:** Demographic characteristics of student leaders who participated in the needs assessment survey

**Respondent Demographic**	**Students (n=36)**	
	n	% (Valid)
*Year of Study*		
First year UG	7	20.6
Second year UG	2	5.9
Third year UG	10	29.4
Fourth year UG	5	14.7
Fifth year UG	1	2.9
Sixth year UG	1	2.9
Taught PG	1	2.9
PhD/Masters by Research	3	8.8
Other	4	11.8
*Mature Student*		
Yes	15	42.9
No	20	57.1
*Field of Study*		
Arts and Humanities	3	8.8
Business	2	5.9
Creative Arts	2	5.9
Engineering	3	8.8
Health Science	21	61.8
Law	1	2.9
Science	2	5.9
*Gender Identity*		
Female	24	70.6
Male	7	20.6
Nonbinary	3	8.8
*Gender identity same as sex at birth*		
Yes	31	91.2
No	3	8.8
*Sexual Orientation*		
Asexual	1	3.0
Bisexual	7	21.2
Gay	1	3.0
Heterosexual/straight	19	57.6
Queer	1	3.0
Other	4	12.1
*International Student*		
Yes	16	47.1
No	18	52.9
*Ethnic Identity*		
Arab	2	5.9
Asian or Asian Irish - Indian/Pakistani/Bangladeshi;	2	5.9
Black or Black Irish - African;	1	2.9
White - Irish	18	52.9
White - any other White background	5	14.7
Other	6	17.6

Table 1 summarises the demographic characteristics of the survey respondents (n=36) in the needs assessment survey. Variables assessed include age, gender, race/ethnicity, educational background, and field of study. Data are presented as the number of participants (n) and the corresponding valid percentage (%) for each category represents the percentage of responses for a category calculated only from the total number of non-missing (valid) cases.

**Table 2:** Situations highlighting the need for gender equality leadership skills

Group think and herd mentality	*“One of the toughest places is for example, you know, campus groups, campus social groups, um, where there’s a concentration of young people. They tend to be very aggressive.” (P13)* *“I think that it would be almost impossible for someone in that position to call out something like that [transphobia] without bearing the brunt of more ridicule. I think that it’s hard enough for a straight white man to call out misogynistic behaviour. So, for someone who doesn’t fit into that rigid stereotype, I think it’s almost impossible. They’d be like, “Oh no, I don’t want to be a party pooper or whatever.” So, even though they can recognize that the behaviour is wrong and they wouldn’t engage in it themselves, they still will sit back and watch it happen or allow it to happen because that’s easier than yourself out there and being like, “No, this is wrong.” Because you’re just seen as being dry or, not fun or boring or whatever, if you’re intervening in all this banter.” (P21)*
Power dynamics and hierarchies	*“Talking back or standing up to superiors is going to negatively affect your career because they write your letters of recommendation. They write your evaluation. I feel that that intimidation of being seen as the bossy medical student*,* or the adamant medical student reflects poorly. There’s the fear of reflecting poorly on you. And then later on being*,* if you’ve them as an examiner or something later down the road*,* they might remember you.” (P7)* *“Consultants make sexist comments all the time. But students and you know*,* SHO’s* [senior house officer] *and reg’s* [registrars] *are quite hesitant to say anything because of the hierarchy*,* right? So maybe having an education of one*,* maybe how to*,* one*,* if you are the target*,* how do you deal with it? How do you support yourself and how do you manage that*,* and also how to go about dealing with it? Who do you bring that to?” (P14)*
Platform available to student leaders to influence change	*“I think student unions can have … They can be and often have more or access to the right room at the right time. Like they might be in certain meetings that other students wouldn’t have access to or have more conversations with very senior people and institutions. … So*,* I think there’s probably really practical skills on like*,* if you want to raise an issue in your institution*,* what is a kind of professional*,* helpful way to do that?” (EI2).*

Table 2 presents selected examples from our data in which student participants (P) and expert interviewees (EI) identified real-life scenarios demonstrating the need for gender equality leadership skills. These situations encompass a range of professional and educational contexts and informed the context for our scenario developments.

**Table 3:** Competency-based framework

Domain	Competency	Description
Knowledge	**What is equality?**	Equality of opportunity and equality of outcome
	**What undermines equality?**	Misogyny
		Subjects such as gender equality and topics specific to women are considered niche and non-essential
		Male avoidance of subjects such as gender and gender equality
		Stereotyping
	**Promoting gender equality**	Representation and role models
Desire	**Self-Awareness**	Awareness of one’s own implicit bias
		Reflection
		Privilege
	**Openness and Creativity**	Openness to learn
	**Self-confidence**	Courage
	**Responsibility**	Motivation
Ability	**Communication**	Active listening
		Story telling
		Difficult conversations
		Conflict resolution
	**Emotional Intelligence**	Empathy
		Compassionate kindness
		Person-centred
	**Interpersonal skills**	Understanding what motivates people Stakeholder management
		Ability to create a sense of psychological safety
		Engaging people
		Leadership
		Awareness of confidentiality and boundaries

Table 3 presents the competency-based Knowledge Desire Ability (KDA) Framework, derived from our needs assessment. This framework identifies critical competencies needed for advancing gender equality leadership. The competencies are organised into three domains: Knowledge (understanding gender biases), Desire (commitment to equality and motivation to challenge inequities), and Ability (advocacy and effective communication). The framework guided curriculum development and learner evaluation.

**Table 4:** Knowledge Desire Ability framework development – Supporting quotes from student focus groups and expert interviews

Knowledge	Value of knowledge & being informed	*“The most important thing is to be informed, have your facts straight. There’s so much misinformation out there, so I would encourage anybody who wants to influence or wants to change things, be very clear on what you want to communicate.” (EI5)*
	Understanding concepts; gender diversity and gender equality	*“When I think of gender equality*,* I don’t just think of man and woman. That’s one facet of it*,* but I think also considering gender diverse people*,* gender fluid people*,* trans people as well as part of that umbrella.” (P15)*
	Defining and understanding misogyny, e.g. microaggressions*	*“A lot of people will find it harder to recognize what a microaggression is if they haven’t actually experienced what the microaggression is. Or learning how to recognize what a microaggression may come forth as…What might be a microaggression to them*,* I might not recognize because I haven’t had the exposure to it or I don’t know what it may look like.” (P3)*
Desire	Self-awareness of our biases	*“Good intentions aren’t enough. We need things to help protect ourselves from these biases and cognitive shortcuts that*,* ‘hey*,* that’s just the way our brains are wired’. And people who are part of the stereotyped group also do that. And so*,* we need to figure out ways to help our brains do what our brains just don’t do on their own with good intentions.” (EI7)* *Challenging your own preconceptions*,* catching yourself thinking things and thinking*,* “Why am I thinking that?” (EI6)*
	Reflection	*“The skill of reflection*,* I think that’s a really core skill for leaders*,* and we would always have a session on building your reflective practice as a leader… think about what went well*,* what part did my style as a leader play in that and where things didn’t go as well as I would have liked? What might I do differently next time?” (EI3)*
	Self-awareness of privilege	*“In history the conversations have always been all-male and in particular*,* at least in Western culture*,* all-male white groups. So*,* I think that with regards to EDI conversations*,* I’m very happy to give my two cents*,* but I also realize my two cents is not as valuable necessarily in this context as my listening to other people’s two cents.” (P1)*
	Openness and creativity	*“Accepting imperfection and forgiving mistakes – your own and others’.” (EI9)* *“Open-mindedness*,* I think*,* is another key trait*,* and having a growth mindset where you’re willing to learn from other people and you’re willing to be corrected and not penalize people for that.” (EI6*)
	Self-confidence	*“I’m [nationality – Asia]. So*,* in my culture*,* it’s expected that women should be quiet and shouldn’t stand up for themselves. So*,* I think coming from that background and growing up like that*,* I haven’t had a lot of confidence*,* so I’ll just speak for myself that sense. So*,* I don’t know. I think it’s still useful to teach people how to be confident.” (P23)* *“Resilience and just not being afraid to put your head above the parapet a little bit.” (EI5)* *“Courage is a really important trait because I think it can be difficult to be the person who keeps calling out things that shouldn’t happen.” (EI6)*
Ability	Communication	*“You have to listen to people because that will help you and that skill is hugely important.” (EI5)* *“Having the correct vocabulary and also having the skill and the comfort level to be able to ask what that vocabulary is if you don’t know what it is.” (P6)*
	Empathy and emotional intelligence	*“I think emotional intelligence is an important skill*,* particularly empathy*,* and I think so much in this area is around being able to empathize with the experience of the other and what it feels like to be in different positions of power or lack of power and what that will feel like.” (EI3)*
	Interpersonal skills	*“Being able to create a sense of psychological safety… And if you are wrong*,* that someone feels comfortable to say*,* ‘Well*,* actually I know you’re the CEO*,* or you’re the leader here*,* but I don’t agree with you.’ And that those robust discussions are not seen as a negative thing*,* but as something that will help you and the organization grow.” (EI8)* *“People are at the centre of everything and it’s how you work with people*,* how you engage with people*,* how you bring them along.” (EI5)*

Table 4 presents supporting quotes from student focus groups and expert interviews used in the development of the Knowledge Desire Ability (KDA) Framework. The quotes illustrate key insights from participants (P) and expert interviewees (EI), providing context and validation for the competencies identified within each domain of the framework. These perspectives underscore the importance of addressing gender equality leadership skills in medical education and clinical practice.

*Microaggressions are the everyday verbal, non-verbal, and environmental slights, snubs, or insults, whether intentional or unintentional, which communicate hostile, derogatory, or negative messages to target persons based solely upon their marginalised group membership (Sue et al. [51]).

**Table 5:** Summary of each LIBRA scenario

Scenario	Themes	Context	Primary Learning Objectives
1	Microaggressions; Allyship; Gender roles; Self-efficacy; Imposter syndrome	Internship (or practice placement)	**Knowledge**: Student will be able to identify when micro-aggressions occur. **Skills required for successful intervention**: Student will demonstrate empathy and interpersonal skills in their discussion with a peer student. Student will communicate appropriately and professionally with supervisor, with an aim to facilitate greater equality between the supervisor and fellow student. **Attitudes required for successful intervention**: Student will demonstrate motivation and a sense of responsibility to intervene.
2	Gender stereotyping; Implicit bias; Gender-based role allocation; Addressing gender inequality among peers	Group project	**Knowledge required for successful intervention**: Student will recognise where gender-based stereotyping has occurred. **Skills required for successful intervention**: Student will employ active listening skills to understand peer’s experience Student will communicate respectfully and confidently to address the gender inequality at play Student will demonstrate an ability to empathise with both perspectives **Attitudes required for successful intervention**: Student will exemplify courage to address the gender inequality at play
3	Power dynamics; Managing uncomfortable conversations; Implicit bias	Lecture	**Knowledge required for successful intervention** Student will identify where implicit bias leads to gender inequality **Skills required for successful intervention** Student will communicate respectfully to advocate for a solution Student will demonstrate empathy to show that they understand the other’s perspective Student will employ appropriate communication skills to address the gender inequality at play, recognising that a positive outcome may not be achievable. **Attitude required for successful intervention** Student will demonstrate confidence in advocating for a solution with classmates and with a lecturer, while remaining appropriate and respectful.
4	Gender diversity; Allyship; Leadership responsibilities	Student Society (2 Committee members)	**Knowledge required for successful intervention**: Student will demonstrate an understanding of gender diversity and different experiences of gender **Skills required for successful intervention**: Student will employ active listening skills to understand and explore others’ perspectives. The student will use empathetic and non-judgemental communication to explain how their behaviour is resulting in gender inequality. Student will demonstrate an ability to collaborate with others to find a solution, addressing the gender inequality present. **Attitude required for successful intervention**: Student will exemplify an attitude that is open to continually learning and accepting it’s ok not to have all the answers.
5	Sexual harassment Bystander intervention	Party	**Knowledge required for successful intervention**: Student will develop an awareness and understanding of the strategies available to intervene as an active bystander (5 Ds – Direct; Distract; Delegate; Delay; Document) Student will understand the importance of safety in the context of bystander intervention **Skills required for successful intervention**: Student will apply bystander intervention strategies in practice (5 Ds – Direct; Distract; Delegate; Delay; Document) Student will demonstrate an ability to communicate calmly and respectfully with their peers with an aim to cease the inappropriate behaviour **Attitudes required for successful intervention**: Student will exemplify courage to ‘go against the grain’ by actively aiming to cease the inappropriate behaviour

Table 5 presents a summary listing of the theme, context and learning outcomes for each of the five scenarios; further details of each scenario are provided in Appendix B.

**Table 6:** Demographic characteristics of learners and simulated participants in the LIBRA pilot

Participant Demographic	Students (n=19)		SPs (n = 8)	
	n	%	n	%
*Age*				
20-24 years	9	47.4	6	75.0
25-29 years	9	47.4	1	12.5
30-34 years	1	5.3	1	12.5
*Gender Identity*				
Female	10	52.6	3	37.5
Male	5	26.3	4	50
Nonbinary	3	15.8	1	12.5
Other	1	5.3	0	0
*Gender identity same as sex at birth*				
Yes	16	84.2	7	87.5
No	3	15.8	1	12.5
*Sexual Orientation*				
Bisexual	1	5.3	1	12.5
Gay	3	15.8	1	12.5
Heterosexual/Straight	9	47.4	5	62.5
Queer	5	26.3	1	12.5
Prefer not to say	1	5.3	0	0
*Ethnic Identity*				
Arab	1	5.3	0	0
Asian or Asian Irish - Chinese	5	26.3	1	12.5
Asian or Asian Irish - Indian/Pakistani/Bangladeshi	4	21.1	0	0
Black or Black Irish - African	1	5.3	1	12.5
White - Irish	5	26.3	5	62.5
White - any other White background	3	15.8	1	12.5
*Mature Student*				
Yes	10	52.6	N/A	N/A
No	9	47.4		
*Year of Study*				
First Year UG	1	5.3	N/A	N/A
Second Year UG	5	26.3		
Third Year UG	2	10.5		
Taught PG	7	36.8		
PhD/Masters by Research	2	10.5		
Other	2	10.5		
*Field of study*				
Arts and Humanities	3	15.8	N/A	N/A
Business	1	5.3		
Engineering	2	10.5		
Health Science	11	57.9		
Law	1	5.3		
Science	1	5.3		

Table 6 presents the demographic breakdown of learners and simulated participants (SPs) involved in simulation-based interventions. The table includes key demographic variables such as age, gender, and educational background, demonstrating the diversity of the participants, which was broadly representative of the student leadership body in the partner HEIs.

**Table 7:** Self-reported pre- and post-intervention learner confidence in gender-enhancing skills

**Stem Question:** **“I am confident in my ability to…”**	Pre-Event %	Post-Event %
	(n=16)	(n=12)
*** Understand and define gender equality principles ***		
Agree/strongly agree	81 (13/16)	92 (11/12)
Neutral/do not know	13 (2/16)	0
Disagree/strongly disagree	6 (1/16)	8 (1/12)
*** Communicate gender equality principles to others ***		
Agree/strongly agree	81 (13/16)	92 (11/12)
Neutral/do not know	13 (2/16)	0
Disagree/strongly disagree	6 (1/16)	8 (1/12)
*** Be actively aware of gender in my own work and planning***		
Agree/strongly agree	75 (12/16)	92 (11/12)
Neutral/do not know	19 (3/16)	0
Disagree/strongly disagree	6 (1/16)	8 (1/12)
*** Be actively aware of gender when working in a group ***		
Agree/strongly agree	88 (14/16)	92 (11/12)
Neutral/do not know	0	0
Disagree/strongly disagree	13 (2/16)	8 (1/12)
*** Be actively aware of gender when socialising ***		
Agree/strongly agree	88 (14/16)	92 (11/12)
Neutral/do not know	13 (2/16)	0
Disagree/strongly disagree	0	8 (1/12)
*** Identify gender inequalities in my academic/departmental/field ***		
Agree/strongly agree	81 (13/16)	92 (11/12)
Neutral/do not know	19 (3/16)	0
Disagree/strongly disagree	0	8 (1/12)
*** Identify gender inequalities in my social circles ***		
Agree/strongly agree	81 (13/16)	92 (11/12)
Neutral/do not know	19 (3/16)	0
Disagree/strongly disagree	0	8 (1/12)
*** Challenge gender inequality in my own thinking ***		
Agree/strongly agree	62 (9/16)	83 (10/12)
Neutral/do not know	25 (5/16)	0
Disagree/strongly disagree	13 (2/16)	17 (2/12)
*** Challenge gender inequality among my close friends ***		
Agree/strongly agree	75 (12/16)	83 (10/12)
Neutral/do not know	12.5 (2/16)	0
Disagree/strongly disagree	12.5 (2/16)	17 (2/12)
*** Challenge gender inequality among my peers ***		
Agree/strongly agree	75 (12/16)	68 (8/12)
Neutral/do not know	6 (1/16)	17 (2/12)
Disagree/strongly disagree	19 (3/16)	17 (2/12)

Table 7 presents the percentage of learners who completed the pre- and post-event confidence questionnaires before (n=16) and after (n=12) the intervention. A 6-point Likert scale was used to measure confidence levels. Data are expressed as percentages, with actual numbers of respondents shown in brackets.

**Table 8:** Free-text feedback on the simulation-based intervention

Feedback Theme	Student Comments
Relevance of Scenarios	*“The topics you guys picked actually happen in real life.”*
Application of Training	*“Every aspect of life really. I kept trying to apply it to my medical teaching, but it’s so much wider than that.”* *“Everyday. Gender-based injustices and discrimination happen every day and everywhere… I will be much more capable of identifying these situations.”* *“In every possible area… it’s harder to challenge them in real life, but at least I got to practice how to tackle the situations.”*
Effectiveness of Training Format	*“Being able to observe and analyse the scenarios and interactions between SPs and participants, but most importantly, the discussion that followed after, which really stimulated and challenged my thinking.”* *“The debriefs were very useful, and showed the thinking behind the actions… It was a place to explore and observe.”* *“The training was very interactive and gave everyone the space to be themselves.”*
Suggestions for Improvement	*“Make it more inclusive, like show male gender being disadvantaged and show the stereotypes that come in from intersectionality like race, colour, ethnicity, which adds to gender inequality.”*

Table 8 summarises the qualitative feedback provided by the participants on the simulation-based intervention. The free-text responses capture learners' perspectives on the effectiveness, strengths, and areas for improvement in the intervention. This feedback was used to refine between iterations of the programme and ensure alignment with learners' educational needs.

## Appendix B

### LIBRA programme resources (View only Hyperlink)

#### Folder 1: LIBRA Introduction & Contents

1a. LIBRA Introduction.

1b. LIBRA Overarching Learning Outcomes.

#### Folder 2: Simulated Scenarios

The subfolders corresponding to each of the five scenarios are as follows:

- Full scenario outline.
- Scenario introduction and debrief material.
- Scenario script for the simulated participants (SPs).

#### Folder 3: Simulated Persons Training

3a. Guidelines - LIBRA SP Training.

3b. SP Invitation Email Template.

3c. SP Online Training Presentation.

3d. SP Training - Co-creation of SP Characters.

#### Folder 4: LIBRA Facilitation

4a. Setup: LIBRA Facilitation.

4b. Pre-intervention Materials.

4c. LIBRA Programme Session Plan.

4d. Introduction Workshop Presentation.

4e. Introduction Workshop Notes.

4 f. LIBRA - Introduction to Simulation-Based Education.

4 g. Closing Discussion.

#### Folder 5: LIBRA Evaluation

5a. LIBRA Evaluation.

5b. LIBRA evaluation forms: Word docs.

5c. LIBRA evaluation forms: QR code examples.

#### Folder 6: Follow-up Materials

6a. Follow-up Email Template.

6b. LIBRA Reflection Tools.

6c. Resources and Support Services Template.
